# Genetic characteristic of mumps virus from 2012 to 2016 and its serum antibody level among general healthy population during 2018–2020 in Jiangsu Province, China

**DOI:** 10.1186/s12879-024-09609-1

**Published:** 2024-07-22

**Authors:** Xiuying Deng, Ying Hu, Peishan Lu, Zhiguo Wang, Hongxiong Guo

**Affiliations:** NHC Key Laboratory of Enteric Pathogenic Microbiology, Jiangsu Provincial Center for Disease Control and Prevention, 172 Jiangsu Road, Nanjing, China

**Keywords:** Mumps virus, Phylodynamic, Molecular surveillance, Gene diversity, Immunity pressure

## Abstract

Mumps is a vaccine-preventable disease with high contagious capability. Its incidence declined rapidly since one dose of mumps vaccine was introduced into Expanded Program of Immunization (EPI) in 2008 in China. Nonetheless, the outbreaks of mumps remain frequent in China. Here we aim to assess herd immunity level followed by one-dose mumps ingredient vaccine and to elucidate the genetic characteristics of mumps viruses circulating in the post vaccine era in Jiangsu province of China. The complete sequences of mumps virus small hydrophobic(SH) gene were amplified and sequenced; coalescent-based Bayesian method was used to perform phylogenetic analysis with BEAST 1.84 software. Commercially available indirect enzyme-linked immune-sorbent IgG assay was used for the quantitative detection of IgG antibody against mumps virus. Our results show that genotype F was the predominant mumps viruses and belonged to indigenous spread, and most of Jiangsu sequences clustered together and formed a monophyly. The prevalence of mumps reached a peak in 2012 and subsequently declined, which presented an obvious different trajectory with virus circulating in other regions of China. The gene diversity of viruses circulating in Jiangsu province was far less than those in China. The antibody prevalence reached 70.42% in the general population during 2018 to 2020. The rising trend of antibody level was also observed. Although mumps antibody prevalence does not reach expected level, mumps virus faces higher pressure in Jiangsu province than the whole of China. To reduce further the prevalence of mumps viruses, two doses of mumps vaccine should be involved into EPI.

**Supplementary Information:**

The online version contains supplementary material available at 10.1186/s12879-024-09609-1.

## Background

Mumps caused by MuV is a high contagious disease characterized by unilateral or bilateral swelling of the parotid glands [1]. Although most mumps cases are self-limiting, more serious complications such as orchitis, pancreatitis, deafness, meningitis and encephalitis can occur [2]. As a vaccine-preventable disease, the incidence of mumps declined rapidly after a single-dose mumps vaccine was introduced. However, the waning of mumps antibody induced by vaccine was observed in many countries [3–5], and mumps outbreaks are still common in the world [6–8]. In China, the annual incidence of mumps was approximately 24.2/100,000 from 2004 to 2013 [9].

Although MuV only has one serotype, it was designated into 12 genotypes according to WHO’s instructions. The surveillance on the phylogenetic characteristics of mumps virus is helpful to identify geographic and temporal circulation of various genotypes and assesses the transmission pathway [10–12]. In China, molecular surveillance for MuV has been conducted since 1995 [13]. Genotype F is predominant and indigenous. Genotype G was found in 2011 at first, then appeared in four provinces [13]. Genotype K was found only in Guangxi province of China, which was imported from Vietnam in 2018 [14]. Nonetheless, the data describing the genetic characteristic of MuV circulating in China are still limited.

Jiangsu province, located in the east of China, has approximately 80 million people. One-dose of measles-mumps-rubella (MMR) vaccine was introduced into the national Expanded Program of Immunization to inoculate children aged at 18-month since 2007 [15]. Among MMR vaccine, mumps virus is live attenuated and belong genotype A. Although more than 95% coverage of one-dose MMR vaccine reached, the reported annual incidence of mumps ranges from 8.85/100,000 to 21.72 /100,000 in recent decade [16]. In China, mumps vaccine strains includes S79 and Wm84 both of which are derived from JL strain belonging to genotype A. To assess immunization effectiveness and reveal the phylogenetic characteristic of MuV circulating in Jiangsu province, we studied phylogenetic dynamics of MuV circulating in Jiangsu province during 2012 to 2016 and serum antibody level among healthy population during 2018 to 2020.

## Materials and methods

### Specimen collection and virus isolation

Throat swabs were collected from patients with the symptoms meeting mumps diagnosis criteria within seven days after an onset. Patients were from mumps outbreaks or sporadic cases. Isolation and identification of MuV were performed according to the methods previously described [13]. Participants were aged from 1 month to 54 years old. The participants were classified into four groups: 0 ∼ 7 months, 8 ∼ 17 months, 1.5 ∼ 5 years old, 5 ∼ 14 years old, 15 ∼ 24years old, over 25 years old, and rural migrant-workers over 15 years old. From 2018 to 2020, a total of 3131 healthy subjects were enrolled. The number of specimens among each group was 504, 525,174,524,482,727, and 195, respectively. The 2 ml of whole blood sample was collected into 5 ml vacuumed tube with EDTA-K3, and then centrifuged at 3000 rpm for 15 min to isolate serum. The serum specimens were transferred to Jiangsu Provincial Center for Disease Control and Prevention Measles\Rubella Net Laboratory to measure mumps virus IgG concentration. From 2018 to 2020, a total of 3131 healthy subjects were enrolled.

### RT-PCR amplification and sequence determination

Viral RNA was extracted using the QIAamp mini viral RNA extraction kit(Qiagen, Germany) according to the instructions provided by the manufacturer. The entire *SH* gene was amplified by RT-PCR according to the method previously described [17]. PCR products were sent to sequence in Shanghai Sangon Biotech Co., Ltd. The quality of sequences were checked for their validity.

### Phylogenetic analysis

Phylogenetic tree of *SH* gene segments with the length of 316 bp was constructed with MEGA11.0 by using the neighbor-joining method, The stability of the nodes was assessed by using maximum likelihood with a bootstrap value of 1,000 replications. tMRCA of F genotype sequences was estimated using coalescent-based Bayesian method in BEAST v1.8.4 software. The best-fit model for nucleotide substitution was tested in jModelTest v3.7 according to Akaike information criterion (AIC). At last, GTR + I + G (general time reversible model (GTR); proportion of invariable sites(I); gamma distribution(G)) was used. The sequences were partitioned into 3-codon positions. The convergence of parameters was analyzed using Tracer v1.7.1. The effective sample size of each parameter was ensured more than 200. The maximum clade credibility tree was generated using Tree Annotator v1.8.4. Mumps virus population growth rate was estimated using a Bayesian coalescent method implemented in Tracer v1.7.1.

### IgG test

Commercially available mumps virus specific indirect enzyme-linked immune-sorbent IgG assay (Serion ELISA classic for mumps virus IgG, catalog numbers: ESR103G) was used for the detection and qualitative determination of IgG antibody concentration against a mumps virus according to the manufacturer’s instructions. All sera specimens were tested at a single dilution of 1:100. Cut-off value was calculated according to the company’s instruction. Upper cut-off value was 0.587, lower cut-off was 0.439. Samples were considered as having protective ability if geometric mean concentration (GMC) was ≥ 100U/mL, and no protective ability if GMC ˂100 U/mL [18]. The positive rate of population (PRP) is calculated as the formula: PRP = the number of the specimens having protective ability/the number of all tested specimens*100%.

### Sequence data

The sequences of *SH* gene using in this study have been submitted to GenBank with accession number KX690522, KX690523, KX690524, KX690571-KX690573, KX690589-KX690594, KX690602, KX690603, KX690604, KX987600, KX987602, KX987605-KX987610, KX987613-KX987630, KX987631, KX987633, KX987635, KX987636, KX987637, KX987648, KX987649, KY680435, KY680449, KY680454, KY680455, KY680457, KY680458, KY680461, KY680462, KY680479, KY680492, KY680493, KY680495. Reference sequences from other provinces of China are follows: KF022112, KF031053, KF022112, KF022118, KY680444, KX987601, KX690596, KY680442, KX987628, KX690606, KX690583, KX690582, KY680436, KX690599, KX690601, KX690598, KX690582, KX580806, KF022122, KF022165, KX69058, KF031054, KF022133, EU780218, KY680443, KY680440, KF170913, HQ199885, HQ199881, HQ199885. Reference sequences from other countries are follows: LC183904, LC034329, KF477295, KF481689, AB105479, KX223397 M28749, JQ945269, AY681495, JQ034452, AY685920, JQ945270, LC486118, JQ946044. In addition, JQ 945,276 and KF878077 were used as the sequence of F genotype and G genotype, respectively.

### *Statistical analysis*

The differences of mumps virus antibody positive rate of between various age groups and years were analyzed by chi-square test with R package. *P* < 0.05 is considered as significant in statistics.

## Results

### Genotype analysis of isolates obtained from Jiangsu province through 2012–2016

**Fig. 1:**
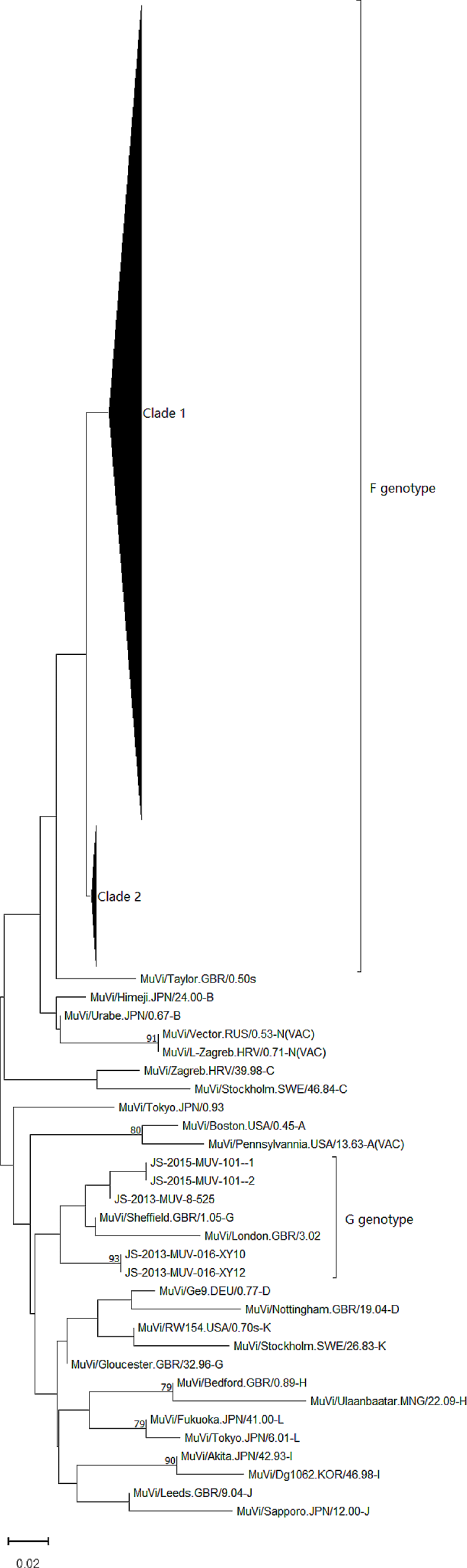
Phylogenetic tree was constructed using using the neighbor-joining method based on *SH* gene

A total of 159 *SH* gene sequences of MuV was obtained. 96.9% of them belonged to genotype F, and 3.1% (5/159) were genotype G(As shown in Fig. 1).

The information of all collapsed sequences in Fig. 1 is provided in supplement 1. Which includes the code of mumps virus isolates, the age and gender of patients, the time and location of virus isolated.

**Fig. 2:**
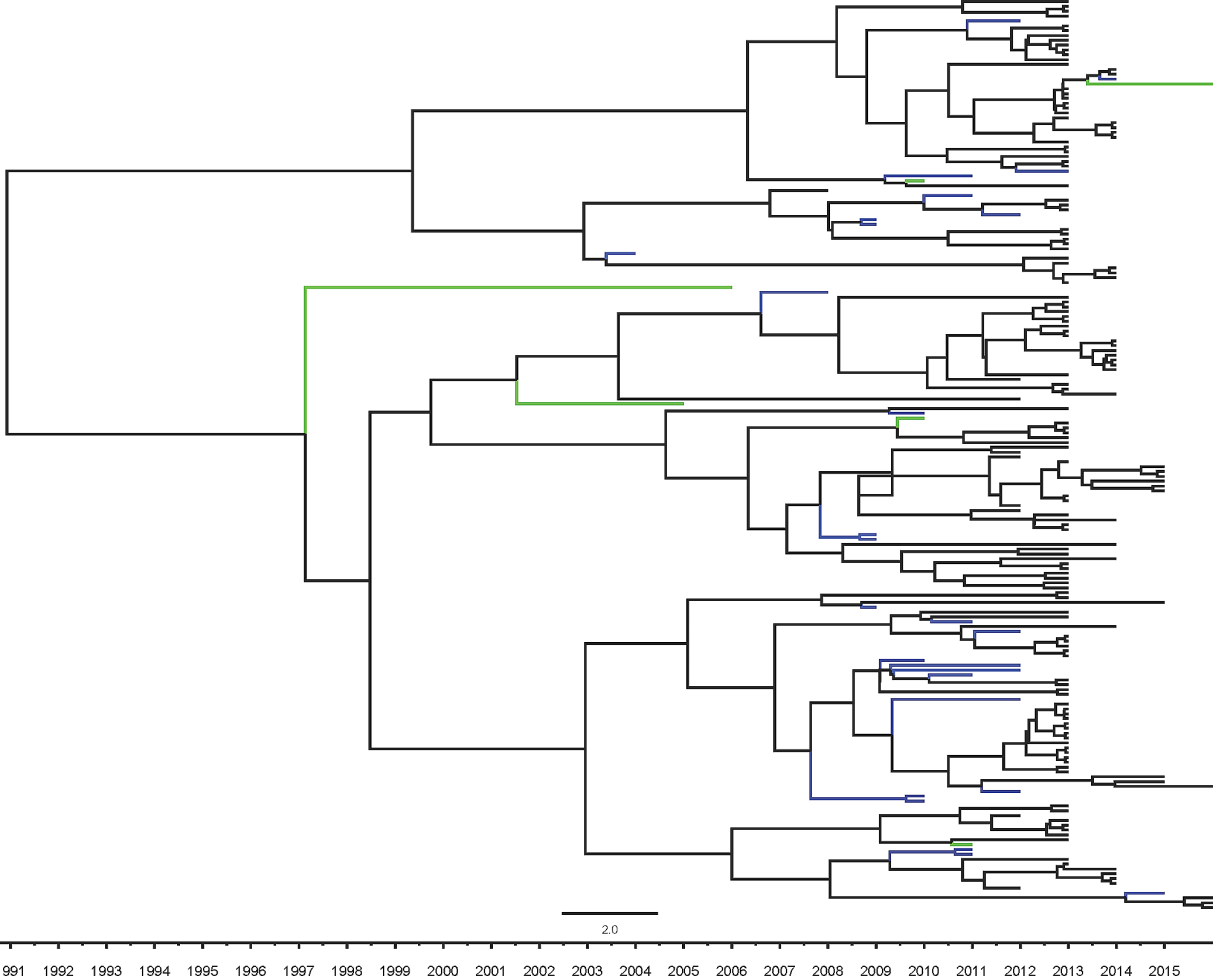
Maximum clade credibility tree of SH of genotype F mumps virus isolated from 2012 to 2016 in the east of China. Black lines indicate sequences isolated from Jiangsu province, blue lines indicate sequences isolated from other provinces of China which include Heilongjiang, Guangdong, Sichuan, Shanghai, Hong Kong, green lines indicate sequences isolated from other countries

**Fig. 3:**
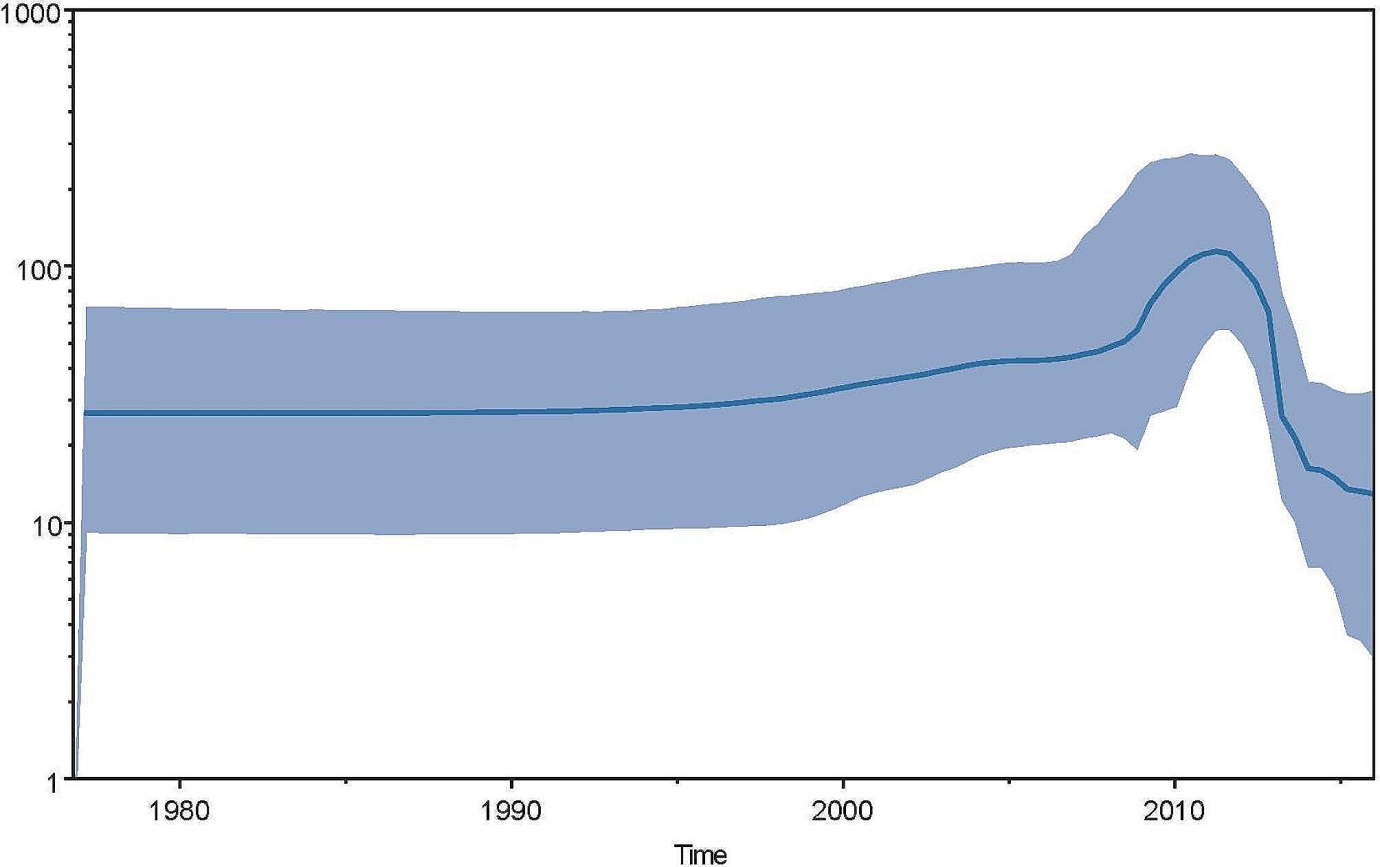
Bayesian skyline plot was estimated to reconstruct mumps virus population in Jiangsu province of China using BEAST 1.84. The x-axis is the time in units of years and the y-axis is equal to the effective population size. The thick solid line is the mean estimates; the 95% HPD credible region is shown as saddled areas

As shown in Fig. 2, most of F genotype sequences circulating in Jiangsu province were clustered together, and formed several independent monophyly. The gene difference of them is less than 3.5 per 100 nucleotides. The closer sequences to them were isolated from Heilongjiang in the north of China, Guangdong and Hong Kong in the south of China, Sichuan in the west of China, Shanghai and Zhejiang in the east of China, respectively. No sequence was closer to those sequences from foreign countries. The Bayesian skyline plot exhibited the rapid decline of MuV population size from 2012 to 2016(in Fig. 3).

### Genetic distance of the small hydrophobic gene within various genotypes

Genetic distance of *SH* gene within Genotype F is 0.041 ± 0.012(0.029 ∼ 0.053), and that within genotype G reaches to 0.033 ± 0.005(0.028 ∼ 0.038). The greatest amount of nucleotide variation among all genotype G sequences was 4.75% (15/316nt) and the corresponding amino acid variation was 10.53% (6/57nt). The mutation rate per site every year of genotype F circulating in Jiangsu province is 1.138 × 10^− 3^(95%CI:6.302 × 10 − 4 ∼ 1.904 × 10^− 3^).

### Humoral immunity against mumps

**Fig. 4:**
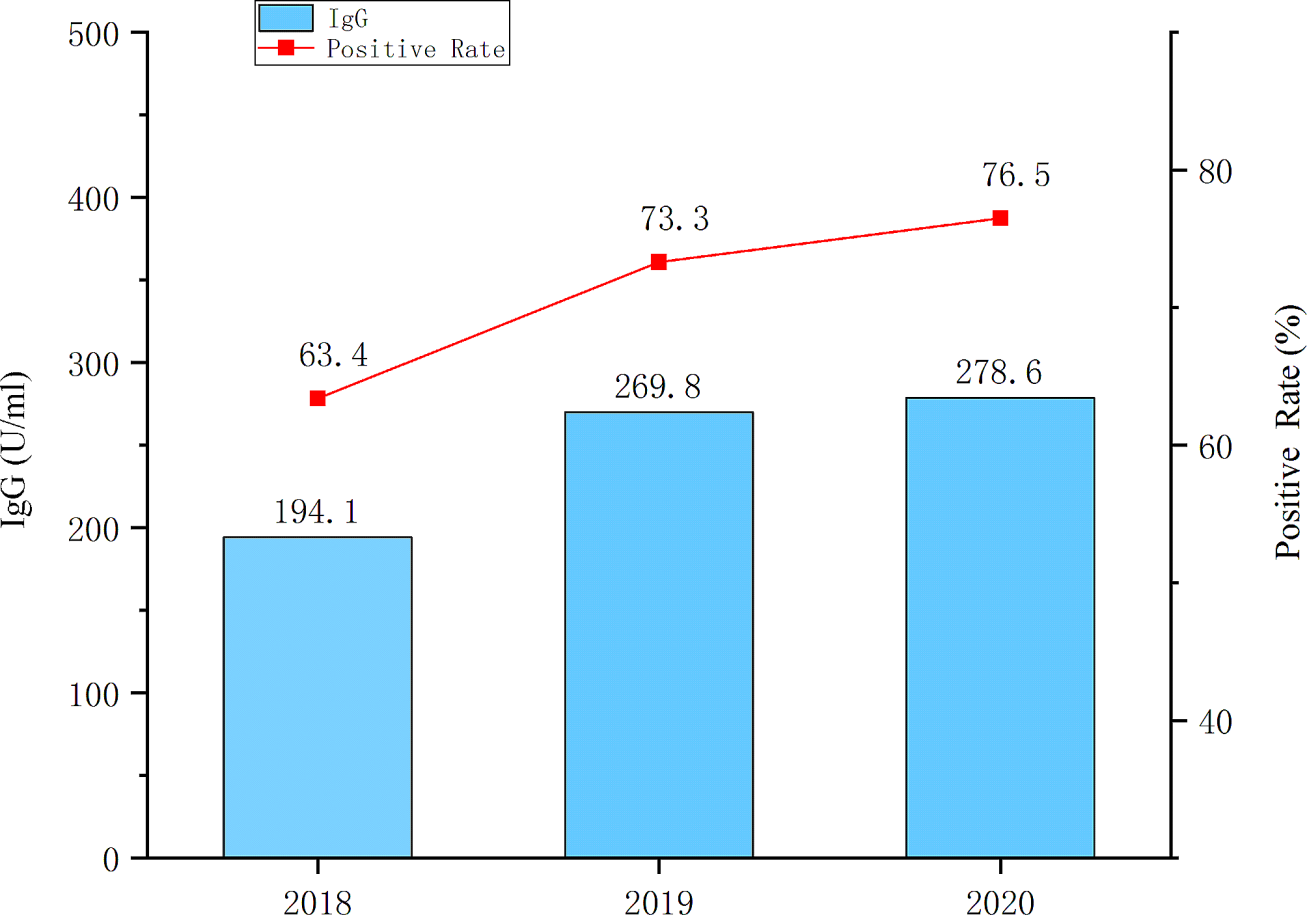
The antibody concentration and positive rate in general population from 2018 to 2020 in Jiangsu province of China. Samples were considered as having protective ability(IgG positive) if geometric mean concentration (GMC) was ≥ 100U/mL, and no protective ability(IgG negative) if GMC ˂100 U/mL

A total of 3131 serum samples (1207 in 2018, 1027 in 2019,897 in 2020, respectively.) were collected from the healthy population living in Jiangsu Province. The subjects were almost equally classified into seven age groups; their ages ranged from less than eight months old to 40 years old. Male accounted for 46.97% while female for 53.03%. As shown in Fig. 4, GMCs were 194.1U/mL in 2018, 269.8 U/mL in 2019, and 278.6 U/mL in 2020, respectively.

**Fig. 5:**
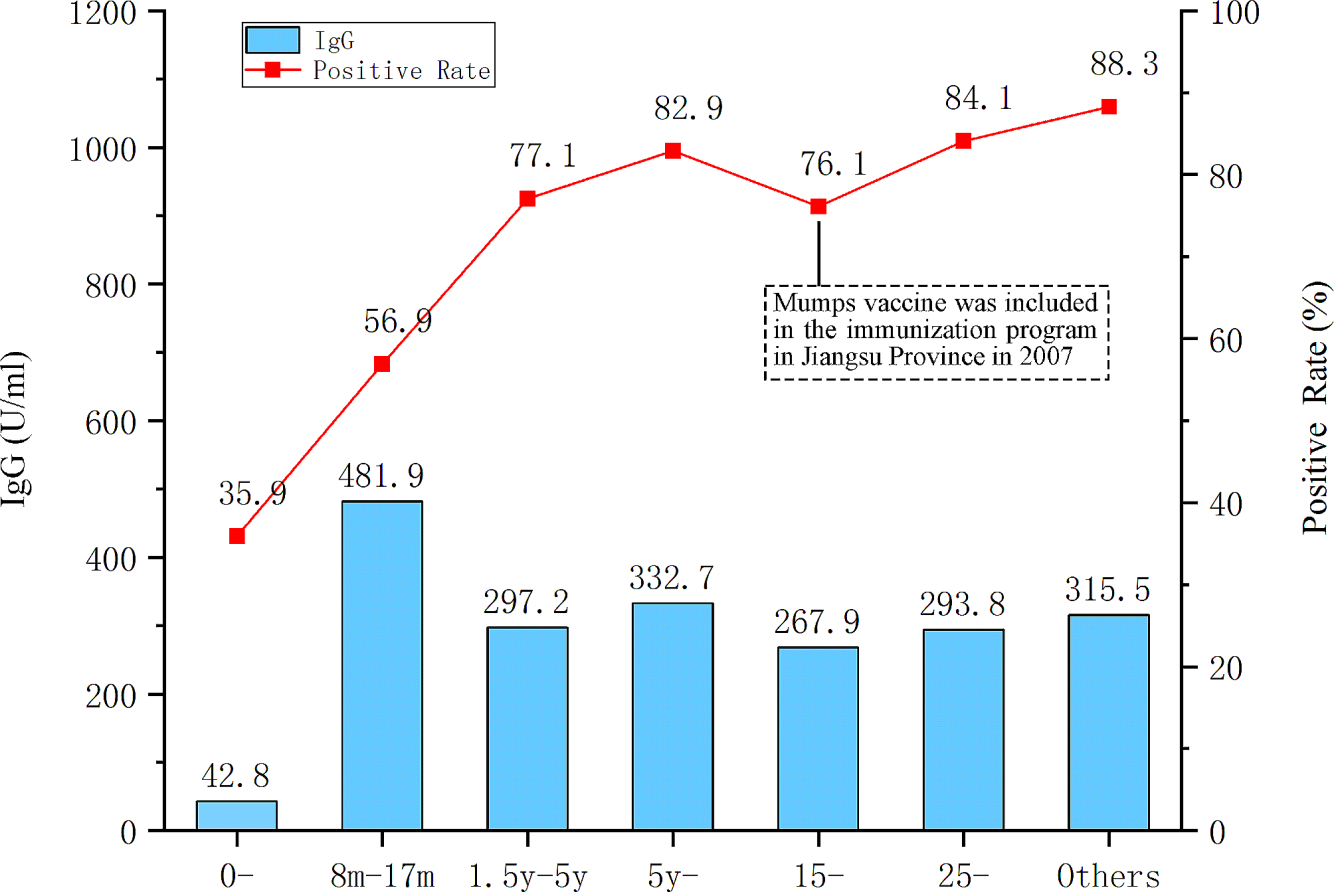
The antibody concentration and positive rate in various age groups

IgG positive rates were 63.4% in 2018, 73.3% in 2019, and 76.5% in 2020, respectively. It shows an increasing trend on IgG positive rates(χ2 = 49.0, *P* < 0.001). When comparing IgG positive rates among health population between 2016 vs. 2017, 2016 vs. 2018, *P* < 0.0001. Although comparing it between 2017 vs. 2018, no significant statistical difference is observed(*P* = 0.55). IgG positive rate was lowest in children under 8-month-old, followed by children at 8 to 17 months old; it rose with the subjects’ age (in Fig. 5). GMC was highest among children aged at 8 to 17 months, and relative stable among population over one and half years old. Antibody positive rate rose with subjects’ age other than population aged from 15 to 24 years old. The obvious difference on antibody positive rate between various age groups was observed((χ2 = 356.9, *P* < 0.001).

## Discussion

Since one-dose of the mumps vaccine program was introduced in China in 2008, mumps incidence declined to 698 per one million population in 2017 [19]. Although mumps cases are routinely reported to National Infectious Disease Surveillance System, no national surveillance proposal for mumps cases is issued until now, which may cause a serious underestimation of mumps incidence. In China, only two genotypes of mumps viruses are circulating [13], in which genotype F is indigenous and predominant [20, 21]. Genotype G MuV was first detected in 2011 in Fujian, Hongkong, and Liaoning, followed by in Jiangsu province in 2013. In this study, we found that more than 95% of sequences circulating in Jiangsu province clustered together, and which suggested that Genotype F MuV circulating in Jiangsu province was indigenous. The sequences of genotype G circulating in Jiangsu were phylogenetically closer to those from Heilongjiang and Hong Kong than those from Fujian. Nonetheless, it was not clear where genotype G circulating in Jiangsu was imported from.

Phylogenetic dynamic analysis showed that the declined trend of the number of population size and gene diversity of mumps virus from 2012 to 2016(Fig. 2). Moreover, both the diversity of *SH* gene of genotype F mumps virus circulating in Jiangsu and their substitution of a nucleic acid site per year are far less than those from other regions of China and other countries [12, 13]. It suggested that mumps virus spreading in Jiangsu is under a higher immunity pressure which partially interrupts the prevalence and evolution of mumps virus, although this pressure may be not enough to interrupt mumps virus epidemic as well as measles herd immunity in most of the regions [22]. Previous study showed that effective population size of mumps virus rose in whole China during 2012–2015 based on skyline plot analysis [23]. It indicates that mumps epidemic status diversifies in various regions of China again [13].

Both seroprevalence rate of mumps IgG antibody and GMCs rosed from 2018 to 2020. It exhibited mumps herd immunity increased among healthy population in recent years. Which is similar to the trajectory of mumps seroprevalence of IgG antibody at the same time in Shanghai metropolitan [23]. However, the seroprevalence rate of mumps IgG antibody is significantly lower in Shanghai (57.37%) than in Jiangsu Province (70.42%). We also found that seroprevalence rate of mumps IgG antibody among various age groups was increased over ages. Which is consistence with those in Shanghai metropolitan [23]. Previous studies show the waning of antibody Levels and avidity even in twice-MMR–vaccinated individuals [3, 24]. Interestingly, the seroprevalence rate of mumps IgG antibody does not decline with age in the studies of ours and the mentioned above. It implies that higher mumps prevalence than the reported and lower surveillance sensitivity may exist, and also reveals that the necessity of the least two-doses of mumps vaccine in EPI(Figure 4). Especially, antibody level of mumps virus in serum rose with age before the first dose vaccine administrated. It implies that the prevalence of mumps is common among children less than 18-month age and advancing vaccination age is necessary. Furthermore, serum mumps antibody level rose with age among the subjects aged over 15 years old, most of them did not receive mumps vaccine and likely ever had mumps virus infected. In general, wild virus infection often induces more persistently immune response compared with the vaccine virus.

In China, although coverage of 95% MMR is required by government, it is difficult to reach this goal in the whole country. Which caused the different immunity pressure after mumps outbreak or the importation of a nonindigenous virus [25–27]. For example, Jiangsu province, as one of the developed regions in China, reaches 98% in the coverage of MMR in recent years. As shown is Fig. 5, migrant workers over 15 years old from other provinces of China have higher antibody level. Which supports that coverage of MMR in Jiangsu province is higher than that in lots of provinces of China. After genotype G imported into Jiangsu province, only cause sporadic mumps cases, then disappeared rapidly. In contrast, genotype K mumps viruses were imported into Guangxi province from Vietnam in 2016, which caused infection and diseases in thirteen individuals [14]. The following epidemiology investigation revealed that none of them had received mumps vaccine [14].

There is a limitation that the exact mumps incidence is not available. Although mumps is a notifiable infectious diseases, no surveillance protocol is issued up to now in China. Therefore, it is common that mumps incidence based on the data from the National Notifiable Disease Reporting System has bigger bias with real incidence to some extent.

## Conclusions

As the predominant genotype of mumps virus circulating in Jiangsu province China, genotype F strain is characterized by indigenous spread and reduced diversity of *SH* gene year-by-year. Although antibody prevalence against mumps does not reach expected level, mumps virus prevalence remains facing higher immune pressure in Jiangsu province than the whole China. To further decrease mumps prevalence, two doses of mumps vaccine may be needed in EPI.

### Electronic supplementary material

Below is the link to the electronic supplementary material.

Supplementary Material 1

## Data Availability

All data are available from corresponding author by email.
